# Research Progress on the Mechanism of the Antitumor Effects of Cannabidiol

**DOI:** 10.3390/molecules29091943

**Published:** 2024-04-24

**Authors:** Li Ma, Mengke Liu, Chuntong Liu, Huachang Zhang, Shude Yang, Jing An, Guiwu Qu, Shuling Song, Qizhi Cao

**Affiliations:** 1 Department of Immunology, School of Basic Medical Sciences, Binzhou Medical University, Yantai 264003, China; 17275466132@163.com (L.M.); lmk04160916@163.com (M.L.); 19550997606@163.com (C.L.); zhc1822001852@163.com (H.Z.); 2 Department of Edible Mushrooms, School of Agriculture, Ludong University, Yantai 264025, China; sdyang68@126.com; 3 Division of Infectious Diseases and Global Health, School of Medicine, University of California San Diego (UCSD), La Jolla, CA 92037, USA; jan@ucsd.edu; 4 Department of Traditional Chinese Medicine, School of Pharmacy, Binzhou Medical University, Yantai 264003, China; qu_guiwu@163.com

**Keywords:** cannabidiol, antineoplastic, apoptosis, cell cycle arrest

## Abstract

Cannabidiol (CBD), a non-psychoactive ingredient extracted from the hemp plant, has shown therapeutic effects in a variety of diseases, including anxiety, nervous system disorders, inflammation, and tumors. CBD can exert its antitumor effect by regulating the cell cycle, inducing tumor cell apoptosis and autophagy, and inhibiting tumor cell invasion, migration, and angiogenesis. This article reviews the proposed antitumor mechanisms of CBD, aiming to provide references for the clinical treatment of tumor diseases and the rational use of CBD.

## 1. Introduction

**Figure 1:**
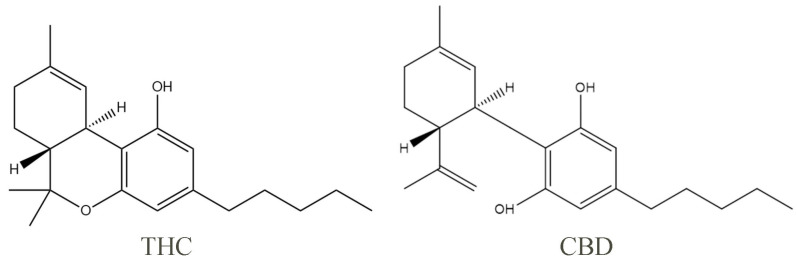
Chemical structural formulas of THC and CBD.

Cannabidiol (CBD) and Δ9-tetrahydrocannabinol (THC), the pure natural ingredients extracted from the cannabis plant, are the two most widely studied phytocannabinoids. One of the properties of pure CBD is that it has pale yellow crystals, which are readily soluble in organic solvents such as ethanol, methanol, and ether and almost insoluble in water. The phenolic hydroxyl group helps to increase the highest occupied molecular orbital value of CBD, so the phenolic hydroxyl group is the structural basis of the antioxidant properties of cannabidiol (Figure 1). With the deepening of research, CBD has also been found to have anti-inflammatory, antitumor, and antiemetic effects, among others [1,2,3,4]. THC is the main psychoactive component of Cannabis sativa, which can act as a partial agonist and bind to the corresponding receptors to exert a variety of physiological effects. Studies have demonstrated that THC has significant analgesic and antiemetic effects, but its clinical application is limited by the psychoactive side effects, such as anxiety and cognitive impairment [5]. Unlike THC, CBD does not harm the mental system, and in fact, it can prevent and reverse the anxiety and addiction caused by the THC-induced overactivation of the extracellular regulated kinase (ERK) pathway [5].

The antitumor activity of CBD has attracted increasing attention and has been demonstrated in various tumor types, such as colorectal, lung, breast, ovarian, and prostate cancers [6,7,8,9]. CBD antitumor mechanisms include the induction of cell cycle arrest and autophagy, the promotion of apoptosis, the modulation of angiogenesis, and the inhibition of tumor cell migration and invasion. In addition, CBD has good synergistic effects with other medicines, and several clinical reports show CBD being used to treat cancer. This article reviews the mechanism through which CBD inhibits tumor growth and metastasis, the synergistic effect between CBD and other antitumor drugs, and the reversal of antitumor drug resistance by CBD. The overall aim is to provide an important reference for the clinical applications of CBD therapy.

## 2. Antitumor Mechanisms of CBD

**Figure 2:**
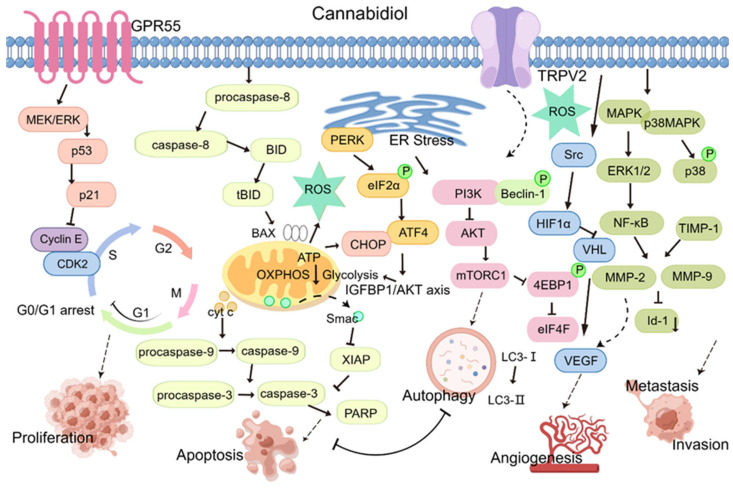
Antitumor activity of CBD (proliferation, apoptosis, autophagy, angiogenesis, invasion and metastasis). CBD via the GPR55 receptor can activate downstream MEK/ERK signaling and activate p53 and p21, leading to a decrease in cell cycle proteins E, and CDK2, resulting in cell cycle arrest. The CBD-induced apoptosis pathway involves pro-caspase-8 recruitment and activation, and activated caspase-8 directly activates effector caspases, such as caspase-3, which initiates the execution process. Truncated BID (tBID) then translocates to the mitochondria and activates the BCL-2 family member Bax. Upon activation, Bax induces mitochondrial outer membrane permeability and the release of proapoptotic mitochondrial contents into the cytoplasm, such as cytochrome c, to recruit pro-caspase-9 to form apoptotic bodies, resulting in the induction of activation of caspase-9 adjacent to the caspases, which cleaves and activates effector caspases. Caspase induces apoptosis. CBD-induced endoplasmic reticulum stress activates PERK oligomerization within the ER membrane, which activates downstream upregulation of ATF4 and CHOP proteins to induce mitochondrial apoptosis in tumor cells. At the same time, endoplasmic reticulum stress inhibits the P13K pathway, leading to a decrease in Akt, which leads to the downregulation of mTORC1 signaling, resulting in autophagy. CBD induces endoplasmic reticulum stress through TRPV2, leading to an increase in ROS production, which also induces endoplasmic reticulum stress. CBD-activated MAPK/ERK and VHL/HIF-1a signaling pathways regulate the expression of VEGF and matrix metalloproteinases MMPs to suppress tumor angiogenesis and inhibit the expression of MMPs. This figure is drawn by Figdraw. ATF4, recombinant activating transcription factor 4; BID, Bcl-2 homology 3 interacting-domain death agonist; CBD, cannabidiol. CHOP, C/EBP homologous protein; ER Stress, endoplasmic reticulum stress; GPR55, orphan G protein-coupled receptor 55; mTORC1, mammalian target of rapamycin complex 1; ROS, reactive oxygen species; TRPV2, transient receptor potential vanilloid 2.

Unlike normal cells, tumor cells have characteristics of unlimited replication, antiapoptosis, continuous angiogenesis, tissue invasion, and metastasis. As a potential tumor treatment drug, CBD exerts its antitumor effects by regulating the multiple processes controlling tumor occurrence and development. This review provides an update on data related to the antitumor effects of CBD (Figure 2).

### 2.1. CBD Regulates the Tumor Cell Cycle

Cell proliferation is regulated by the cell cycle, and blocking the division cycle of tumor cells is an important antitumor mechanism. Eukaryotic cells rapidly synthesize RNA and protein through the G1 phase, which provides material and energy for DNA synthesis in the S phase. The G2 phase assembles materials needed for cell division and prepares the cell for the M phase, or mitosis. Therefore, in addition to overriding the S and M phases to inhibit the proliferation of tumor cells, regulating the G1 and G2 phases is of great significance for cancer treatment. Several studies have proven that CBD treatment can render tumor cells susceptible to G0–G1 phase blockage.

In thyroid and breast cancer cells, CBD exerts its antiproliferative effect by causing cell cycle arrest during the G1/S phase transition, but it has no similar effect in non-neoplastic cells [10]. In pancreatic cancer, CBD inhibits cell cycle progression by binding to the GPR55 receptor and dose-dependently blocking its activity, resulting in the downregulation of the MAPK/ERK signaling pathway, the inhibition of the G1/S transition, a reduction in the DNA synthesis in the S phase, and the suppression of pancreatic cancer cell growth [11]. The cell cycle is regulated by cyclin and cyclin-dependent kinases (CDKs). Cyclin and CDKs can combine to form a heterodimer that promotes the procession of the cell cycle. The complex formed by cyclin E and CDK2 can promote the transition from the G1 phase to the S phase. Zhang et al. [12] studies showed that CBD inhibits the expression of cyclin E and CDK2 in gastric cancer by significantly downregulating the expression of the p53 protein and upregulating the expression of the p21 protein and the ataxia telangiectasia mutant gene, leading to cell cycle arrest in the G0–G1 phase and subsequently exerting an antiproliferation effect. Similar effects have also been reported for glioblastoma cells. CBD exerts its antiglioblastoma effects by specifically regulating the activities of ERK and multiple caspases (caspase-3, caspase-7, and caspase-9), resulting in the arrest of U251 and SF126 cells in the G0–G1 phase. Combined therapy consisting of CBD and THC can significantly enhance this inhibition [13].

### 2.2. Multiple CBD-Associated Pathways Can Induce Tumor Cell Apoptosis

Apoptosis refers to the autonomous and orderly death of cells. It is controlled by genes and is associated with processes that actively remove redundant or differentiated cells that are incompatible with the body, cells that have completed their functions but are no longer used/needed, and cells that are potentially dangerous. Therefore, apoptosis is considered an indispensable regulatory mechanism for the normal development of organisms; however, when out of control, apoptosis leads to various diseases, including tumorigenesis. A defect or obstruction in apoptosis can disrupt the normal proliferation, differentiation, and death programs of normal cells, and this disruption is one of the factors underlying tumorigenesis. At present, the clinical use of radiotherapy and chemotherapy drugs, which are mainly used to induce tumor cell apoptosis, shows efficacy. Recent research results have confirmed that CBD can exert its antitumor effects by inducing tumor cell apoptosis through several processes, including the disruption of mitochondrial homeostasis and the imposition of endoplasmic reticulum stress (ERS).

Mitochondria are the activity center of apoptosis regulation, and they play an important role in the apoptosis pathway [14]. Various antioxidant systems in cells and mitochondria can regulate the production of mitochondrial reactive oxygen species (ROS) to maintain intracellular ROS homeostasis under normal physiological conditions. The disruption of the mitochondrial ROS homeostasis is an important factor in the development of diseases in the body. In breast cancer MDA-MB-231 cells, CBD induces BH3 interacting-domain death agonist (BID) production and translocation, increases cytochrome C release, and ultimately promotes apoptosis by reducing the mitochondrial membrane potential. In addition, CBD treatment can increase the production of ROS. The use of the antioxidant α-tocopherol (TOC) to limit the ROS level can decrease the expression of apoptotic protein induced by CBD, again implicating ROS in apoptosis [15]. Other studies have revealed that CBD activates a caspase cascade in leukemic Jurkat cells, leading to PARP cleavage, the upregulation of NADH oxidase expression, increased ROS production, and finally cell apoptosis [16]. Massi et al. [17] reported that CBD triggers apoptosis in glioma cells by activating caspase-8 and caspase-9 through the early generation of ROS and the depletion of glutathione (GSH).

Another apoptosis trigger is ERS, defined as the accumulation of unfolded or misfolded proteins in the endoplasmic reticulum in response to hypoxia, acidosis, and other stress stimuli. High-intensity and sustained stress responses can induce apoptosis. In colon cancer cells, CBD induces ERS and increases the expression of death receptor DR5 by phosphorylating the double-stranded RNA-dependent protein kinase-like ER kinase-C/EBP homologous protein (PERK-CHOP) while also enhancing tumor cell apoptosis induced by tumor necrosis factor-related apoptosis-inducing ligand (TRAIL) [18]. In addition, CBD dose-dependently induces apoptosis in gastric cancer cell lines (AGS, MKN45, SNU638, and NCI-N87). The molecular mechanism of CBD action involves the induction of ERS and mitochondrial dysfunction by translocating the proapoptotic factor Smac to the cytoplasm to bind to the X-linked inhibitor of apoptosis protein (XIAP), thereby increasing the ubiquitination of XIAP in gastric cancer cells [19].

CBD can also induce tumor cell apoptosis in other ways. Sreevalsan et al. [20] demonstrated that CBD promotes phosphatase-dependent and/or CB receptor-dependent apoptosis in prostate cancer LNCaP and colon cancer SW480 cells. In lung cancer, CBD induced H460 and A450 cell apoptosis through the upregulation of the adhesion molecule ICAM-1 and tumor cell death medicated by lymphokine-activated killer cells (LAK) [21]. Micro-RNA (miRNA), which are short non-coding RNAs, may also be involved in tumor pathogenesis through the regulation of apoptosis [22]. Alharris et al. [23] published the first report showing that CBD can control tumor cell invasion, migration, and apoptosis through miRNAs. CBD can specifically downregulate miRNAhsa-let-7a and increase the expression of caspase-3 and GAS-7 genes. These changes, in turn, activate neuroblastoma (NBL) cell apoptosis.

### 2.3. CBD Induces Tumor Cell Autophagy

In addition to its proapoptotic effects, CBD also demonstrates the capacity to induce autophagy in neoplastic cells. Autophagy, recognized as type II programmed cell death, encompasses cell demise initiated by the breakdown of misfolded proteins or dysfunctional organelles. Autophagy plays a pivotal role in various stages of carcinogenesis, including tumorigenesis, progression, metabolism, and metastasis, thereby exhibiting a dualistic impact on numerous malignancies [24]. During the initial phases of tumorigenesis, autophagy potentially impedes carcinogenesis by eliminating faulty proteins and organelles [25]. Unfortunately, in the advanced stages of cancer progression, particularly when the tumor confronts stressors (e.g., restricted angiogenesis, nutrient deficiency, and hypoxia), autophagy assumes a prosurvival role and instead fosters tumor proliferation, metastasis, and invasion [26]. Cellular autophagy has therefore emerged as a focal point for antineoplastic therapy [27]. Consequently, the precise mechanism through which CBD elicits its antitumor effects through the instigation of autophagy in tumor cells has garnered substantial attention.

CBD induced the aggregation of the autophagy marker LC3-II protein in a human colonic epithelial cell line [28]. During autophagy, the LC3 protein is synthesized and hydrolyzed by ATG4 to produce cytoplasmic LC3-I, which then covalently binds to phosphatidylethanolamine under the action of Atg7 and Atg3. LC3-I is subsequently lipoylated to form LC3-II, which binds to the autophagosome membrane to form an autophagosome [29]. This process has been confirmed in several colorectal cancer (CRC) cell lines [30]. In addition, CBD-induced LC3-II overexpression has been found in glioma cells. However, in these cells, CBD enhanced the early autophagic flux rather than promoting the complete autophagic process. CBD-induced cell death could be blocked by early autophagy inhibitors (wortmannin and LY294002), whereas late autophagy inhibitors (CQ and Baf-A1) promoted cell death [31]. The autophagy process is closely related to apoptosis, and some studies have confirmed that CBD-induced autophagic responses sometimes coexist with apoptosis. One of these responses is associated with Beclin-1, a mammalian autophagy gene that is also a key target for regulating autophagy. Beclin-1 can bind to Bcl-2 family proteins to form a protein complex to regulate autophagy levels [32], and CBD can inhibit the connection between Beclin1 and Bcl-2, thereby increasing the level of autophagy. In breast cancer MDA-MB-231 cells, CBD treatment induced ERS, resulting in the accumulation of LC3-II and triggering autophagic cell death. In this case, a large amount of caspase-3 produced by apoptosis cleaves Beclin-1 and blocks autophagy. At the same time, the cleavage product is transferred to the mitochondria, where it induces apoptosis by promoting Cyt-c release. Interestingly, the dominance of apoptosis and autophagy seems to be determined by the concentration of CBD [15]. CBD also upregulates acute myeloid leukemia (AML)-1 transcription factors, binds to the transient receptor potential Vanilloid-2 (TRPV2) promoter in a PI3K/AKT-dependent manner, and activates TRPV2 to trigger the differentiation of glioma stem cells (GSCs), which activates the autophagy process and inhibits GSC growth [33]. In addition, some studies have demonstrated in ex vivo experiments that the combination of CBD and oxaliplatin treatment of CRC cells can reduce nitric oxide synthase 3 (NOS3) activity, leading to mitochondrial dysfunction-mediated ROS production and excessive ERS, and ultimately induce autophagy, and thus it has an antitumor role [34].

### 2.4. CBD Inhibits Tumor Cell Angiogenesis

Angiogenesis is a dynamic process of endothelial cell proliferation, adhesion, migration, and vascular ring formation that is regulated by both angiogenic mediators and inhibitors [35]. Uncontrolled angiogenesis can cause several disorders, including some immune diseases (rheumatoid arthritis), tumorigenesis, and tumor metastasis. Many studies have supported the possibility that CBD can exert its antitumor effects by inhibiting tumor angiogenesis.

Solinas et al. [36] found that CBD in glioma U87-MG cells significantly interfered with and decreased the protein expression of various regulatory factors involved in the angiogenic process, such as vascular endothelial growth factor (VEGF), matrix metalloproteinase-2/9 (MMP-2/9), matrix metalloproteinase inhibitor-1 (TIMP-1), urokinase-type fibrinogen activator (uPA), etc., but no specific signaling pathway was identified. Among them, the matrix metalloproteinase family (MMPs) can be involved in angiogenesis through the disassembly of the basement membrane and remodeling of the extracellular matrix [37]. In contrast, TIMP-1 is an endogenous metalloproteinase inhibitor that inhibits the activity of MMPs [38]. HIF-1α, as a major regulator of oxygen homeostasis, plays a key role in tumor angiogenesis and metastasis [39]. A recent study has shown that CBD can inhibit breast cancer growth by inhibiting SRC activity, thereby upregulating VHL expression, reducing HIF-1α synthesis in breast cancer cells, and inhibiting angiogenesis [40]. Tumor-associated macrophages secrete EGF and promote angiogenesis [41]. Elbaz et al. [42], in a study on the role of CBD in breast cancer, reported that CBD inhibits angiogenesis, thereby playing an antitumor role. It does so by regulating the tumor microenvironment through the modulation of cytokine production by tumor cells, which results in less recruitment of total macrophages and M2 macrophages to primary and secondary tumor sites.

Although the role of cannabinoids and their analogs in inhibiting angiogenesis has been extensively studied, the antiangiogenic effects of CBD have not been well investigated. In addition to the proven targets of CBD’s angiogenesis-inhibiting effects, a number of small molecules and proteins have emerged that potentially inhibit angiogenesis and are worth exploring. For example, endothelial TRPV4 has been demonstrated in recent years as a key regulator of vascular integrity and tumor angiogenesis, suggesting that targeting TRPV4 may represent a potential new strategy for vascular normalization and cancer therapy [43]. Given that CBD can activate TRPV4, CBD may exert its antiangiogenic effects by targeting TRPV4 [31]. In addition, GPR55, which was shown to affect endothelial cell proliferation by decreasing nerve growth factor (NGF) levels in the TME, can also act as a receptor for CBD action. Therefore, its relationship deserves to be investigated in depth to provide a new approach for inhibiting tumor angiogenesis [44].

### 2.5. CBD Suppresses Tumor Cell Invasion and Migration

The remarkable characteristics of malignant tumors include their capacity for distant invasion and migration of cells. These metastatic cells are also important determinants of mortality among patients with clinical cancers. Research findings substantiate CBD’s capability to inhibit the invasion and migration of various types of tumor cells.

The epithelial–mesenchymal transition (EMT) has garnered extensive attention due to its potential role in transforming benign tumors into aggressive and metastatic malignancies. The EMT has been established as a pivotal process associated with tumor invasion and metastasis. During EMT progression, numerous genes undergo altered expression, including the downregulation of epithelial phenotype-associated proteins (e.g., E-cadherin, keratins, and tight junction protein-1) and the upregulation of mesenchymal phenotype-associated proteins (e.g., N-cadherin, vimentin, and fibronectin) [45]. Targeting EMT-related signals therefore holds promise as a strategy for halting tumor progression. CRC cells treated with CBD show the inhibition of the EMT process due to the disruption of the Wnt/β-catenin signaling pathway. This intervention increases E-cadherin expression and decreases N-cadherin expression, thereby effectively obstructing the Wnt pathway and preventing cancer cell migration [46]. Similarly, in breast cancer cells, CBD demonstrates the capability to restore the epithelial tissue lost due to cell dispersion by facilitating the repositioning of E-cadherin and β-catenin at cell adhesion junctions. CBD also thwarts the nuclear translocation of β-catenin by blocking the IL-1β-induced IL-1β/IL-1RI/β-catenin signaling pathway that regulates migration and progression, one of the factors crucial in driving the EMT [47]. Consequently, CBD might counteract tumor invasion and migration by reversing the EMT. Another target of CBD with the potential to inhibit invasion is the fibrinogen activation system. In that system, plasminogen activator inhibitor type 1 (PAI-1), a member of the serine protease inhibitors, can inhibit fibrinogen activator and interfere with malignant tumor cell invasion [48]. Some studies have confirmed that CBD inhibits PAI-1 expression in lung cancer A549 cells through a cannabinoid receptor 1 (cannabisreceptor1, CB1)/cannabinoid receptor 2 (cannabisreceptor2, CB2)/TRPV1-dependent pathway, which inhibits cell invasion.

MMPs synthesized and secreted by tumor cells can target a variety of proteins in the extracellular matrix (ECM) and degrade the ECM, resulting in tissue structural relaxation and promoting tumor cell invasion and metastasis [49]. CBD inhibits the activation of the EGF/EGFR signaling pathway and the activity of its downstream targets, AKT, ERK, and NF-κB to inhibit tumor cell proliferation and, at the same time, inhibits breast cancer cell invasion and migration through the downregulation of MMP- 2 and MMP-9 secretion, inhibiting breast cancer cell invasion and migration [32]. The transcriptional regulator Id-1 has been reported to be dysregulated in the expression of several types of cancers and is a key determinant of tumorigenesis and metastasis [50]. In breast cancer cells, McAllister et al. [51] showed by analyzing three different classes of cannabinoid compounds that the compound with the most pronounced inhibitory effect on tumor cell proliferation, invasion, and migration was CBD, and further study of the related mechanism of action indicated that CBD could inhibit the expression of the transcriptional regulator Id-1 in breast cancer cells by increasing both p-ERK and ROS, thereby reducing cell proliferation and invasion. Additionally, in glioma cells, CBD plays a similar role, significantly downregulating Id-1 expression and reducing glioblastoma cell invasion after CBD action [52]. In addition, the CBD activation of P38 and MAPK after treatment of lung cancer cell lines A549, H358, and H460 cells induced the expression of TIMP-1, which reduced tumor cell invasion, while in vivo experiments confirmed that CBD had a significant inhibitory effect on lung metastasis in the A549 mouse model [53]. An earlier study using a mouse breast cancer metastasis model also confirmed that CBD reduced the total breast cancer metastasis to the lungs to 75%, inhibited the growth of lung metastases, and showed greater potency in terms of its antitumor invasive ability than in its ability to inhibit the growth of primary tumors [54].

### 2.6. CBD Affects Energy Metabolism in Tumor Cells

One notable characteristic of cancer is the metabolic reprogramming of tumor cells, and the abnormal metabolism in tumor cells can accelerate the malignant progression of cancer [55]. Its main methods include glycolysis, oxidative phosphorylation, amino acid metabolism, fatty acid metabolism, etc. Under normal circumstances, aerobic oxidation is the main pathway through which the body obtains energy. During the period of hypoxia, the body produces energy through glycolysis. Warburg noted that tumor cells are more likely to be powered by glycolysis, even under aerobic conditions [56]. However, glycolysis certainly produces ATP for the vital activities of tumor cells, and it also provides intermediates for protein and lipid synthesis [57], thereby facilitating the growth and proliferation of tumor cells. This indicates that CBD can also exert its antitumor effects by regulating the energy metabolism of tumor cells.

Some studies have shown that CBD significantly reduces the basal cellular respiratory rate and total ATP production, thereby inhibiting the growth of human gastric cancer AGS cells [19]. Similar effects were also observed in neuroblastoma (NBL) cells. CBD retards the growth of NBL cells by inhibiting mitochondrial respiration, although the rate of glycolysis is not significantly changed [23]. To date, few reports have addressed the specific mechanism of the CBD regulation of bioenergy metabolism in tumor cells. Our recent studies have indicated that CBD can inhibit ovarian cancer cell growth by disrupting the LAIR-1-mediated interference of mitochondrial bioenergy metabolism and the PI3K/AKT/mTOR pathway [9]. Shangguan et al. [58] demonstrated that CBD can suppress the growth of hepatoma HepG2 and MHCC97H cells by regulating the ATF4-IGFBP1-AKT axis and decreasing the overall rate of aerobic glycolysis and energy metabolism. Recently, Sun et al. [59] used single-cell RNA sequencing (scRNA-seq) and single-cell ATAC sequencing (scATAC-seq) methods to examine the tumor microenvironment (TME) of mouse MC38 xenograft tumors following the administration of CBD. Their findings indicated that, in macrophages, CBD inhibited oxidative phosphorylation and fatty acid oxidation while promoting glycolysis by suppressing the PI3K-Akt signaling pathway and related downstream target genes. In addition, CBD significantly reduced the number of alternatively activated M2 macrophages and correspondingly increased the number of M1 macrophages, thereby significantly promoting antitumor immunity and suppressing tumor growth in tumor-bearing mice.

Tumor drug resistance remains one of the major challenges limiting successful tumor therapy. Metabolic reprogramming, especially of the glycolytic pathway, shows a close association with malignant drug resistance. A recent study in a transgenic mouse model of prostate cancer confirmed that CBD inhibits the development of hormone-refractory prostate cancer (HRPC) in that model by reprogramming metabolic- and oncogenic-related signals. Mechanistically, CBD inhibits oxidative phosphorylation in enzalutamide-resistant HRPC cells by increasing glycolytic capacity and inhibiting oxidative phosphorylation. This effect of CBD stems from its inhibition of the mitochondrial voltage-dependent anion channel 1 (VDAC1), a key protein in the mitochondrial outer membrane, by regulating the PI3K/Akt/mTOR pathway, which is involved in the control of mitochondrial and cellular functions. The regulation of mitochondrial function and apoptosis [60] is coupled to hexokinase II (HK-II) activity, as cutting off the ATP supply required for the HK-II reaction leads to apoptosis and autophagy. Accordingly, the effects of CBD on cell survival and mitochondrial ATP production were significantly reduced when DIDS, the VDAC1 oligomerization inhibitor, was supplied in combination with CBD [61]. Apparently, the drug resistance of tumor cells can change in response to an alteration in endogenous metabolism, a possibility that provides vibrant new ideas for the development of novel therapeutic agents aimed at overcoming drug resistance.

## 3. Synergistic Sensitization of Antitumor Drugs and Reversal of Drug Resistance

Chemotherapy is a common clinical treatment for many types of cancer, but patients are prone to develop drug resistance during chemotherapy, leading to treatment failure. The active ingredients of many traditional Chinese medicine (TCM) products can reverse chemotherapy resistance in tumor cells. There is evidence that CBD can be applied as an antitumor drug sensitizer in the treatment of malignant tumors and that CBD can effectively reverse tumor drug resistance. Several preclinical studies, including cellular and animal experiments, have examined the use of CBD in combination with antitumor drugs. Below are some cases of CBD combinations tested in different types of tumors.

CBD has a synergistic effect with chemotherapeutic agents. DNA-damaging drugs are still commonly used in the treatment of some malignant tumors, but resistance to these drugs is obvious. Some studies have demonstrated that CBD can increase calcium ion uptake through TRPV2 channels, thereby enhancing the cellular uptake of chemotherapeutic drugs and subsequently increasing the sensitivity of glioma cells to chemotherapeutic drugs, including DNA-damaging drugs (temozolomide, carmustine, or cisplatin) [62]. In addition, Massi et al. [63] demonstrated in ex vivo experiments using glioma tissues and cells that CBD inhibits tumor growth by modulating the lipoxygenase (LOX) pathway and the endogenous cannabinoid system, whereas pretreatment with the 5-LOX-specific inhibitor MK-886 enhanced the antimitotic ability of CBD, suggesting that a combination of CBD and a 5-LOX inhibitor could enhance the effects of CBD.TRAIL acts with the corresponding receptor at the same time but also results in drug resistance, short plasma half-life, and other problems [64]. Kim J.L. et al. [18] found a synergistic antitumor effect of CBD with TRAIL in vitro, which could increase its sensitivity; however, this synergistic effect was not observed in normal colon cells. A recent study showed that combining CBD with photodynamic therapy (PDT) enhanced the production of cytotoxic substances, stimulated the patient’s immune system, and interfered with multiple signaling pathways associated with the regulation of tumorigenesis, drug resistance, and metastasis, thereby preventing the secondary spread of colorectal cancer stem cells and augmenting the therapeutic effect of PDT in colorectal cancer [65]. Mokoena et al. [66] also noted that a combined treatment of CBD and PDT had a strong in vitro killing effect on MCF-7 breast cancer cells, and they proposed that the mechanism might be related to the induction of apoptosis. In hepatocellular carcinoma, cannabidiol promotes cabozantinib-induced tumor cell death by regulating p53 phosphorylation under ERS [67].

Some studies have confirmed that GSCs are significantly resistant to treatment with the conventional antitumor drug carmustine, also called BCNU, as the cell viability of GSCs was only slightly decreased after BCNU treatment. However, when combined with CBD, the cytotoxicity of BCNU was increased due to the induction of mitochondria-dependent apoptosis by CBD, thereby overcoming the high resistance of GSCs to BCNU treatment [33]. ROS are also involved in glioblastoma drug resistance, and CBD treatment of mice bearing GSC tumors greatly increased their tumor ROS levels. This inhibited the survival and self-renewal of the GSC tumor cells and significantly increased the survival of the mice. Combining CBD with inhibitors of antioxidant response genes further enhanced the ability to inhibit tumor growth, invasion, and self-renewal of GSCs [68]. Jeong et al. [34] established oxaliplatin-resistant DLD-1R and Colo205 R colorectal cancer cell lines and showed that the elevated phosphorylation of NOS3 was the key to oxaliplatin resistance. They also verified that the combination of CBD and oxaliplatin could induce the overproduction of ROS through mitochondrial dysfunction, and this further inhibited NOS3 phosphorylation to overcome oxaliplatin resistance. Another in vivo experiment confirmed that the combination of CBD and gemcitabine (GEM, one of the most commonly used drugs for the treatment of pancreatic ductal adenocarcinoma) prolonged survival time by nearly threefold over GEM alone in a mouse model of pancreatic ductal adenocarcinoma [11]. Similarly, a combination of CBD and imatinib, a drug targeted for the treatment of chronic myeloid leukemia (CML), reversed imatinib resistance [69]. Ozmen et al. [70] confirmed by pathohistological examinations that CBD treatment reversed the pathology of congestion, edema, inflammatory cell infiltration, and the loss of epithelial cells in the lungs commonly associated with the use of methotrexate (MTX). MTX is a drug widely used in the treatment of various cancers, but its use can cause adverse effects on organs, especially the lungs.

Extracellular vesicles (EVs), including exosomes and microvesicles (EMVs), and apoptotic vesicles play important roles in the physiological and pathological processes of the body. The release of EMVs in tumors is associated with chemotherapy resistance in cancer cells [71]. Kosgodage et al. [72] demonstrated that CBD significantly reduced the release of exosomes and EMVs in three different cancer cell lines, namely prostate cancer (PC3), hepatocellular carcinoma (HEPG2), and breast adenocarcinoma (MDA-MB-231), and further demonstrated that the modulatory effect of CBD was both dose-dependent and tumor-cell-type-specific and resulted in the increased sensitivity of tumor cells to chemotherapy. In recent years, drug-carrying particle technology has also been applied for the delivery of combinations of antitumor drugs to achieve precise targeting, reduce the risk of metastasis, and reduce tumor resistance [73]. One team that evaluated the synergistic activity of CBD in combination with paclitaxel (PTX) for the treatment of ovarian cancer found that the dose of PTX could be reduced, thereby reducing the toxicity and resistance associated with the drug. Furthermore, PLGA particles could serve as CBD carriers and enhance the antitumor activity of PTX but not of adriamycin and cisplatin. In fact, CBD showed antagonistic effects [74]. Another study showed that integration of CBD into a bionic carbon monoxide nanocomplex (HMPOC@M) increased autophagic flux and promoted cancer cell death through the activation of the class III phosphatidylinositol 3-kinase (PI3K-III)/BECN1 (Beclin-1) complex. Meanwhile, in vivo experiments demonstrated that HMPOC@M+laser treatment strongly attenuated liver and lung metastasis and inhibited tumor growth by downregulating VEGF and MMP9 proteins [75].

## 4. Clinical Application for the Prevention and Treatment of Tumors

Although many in vitro and preclinical in vivo studies have explored the antitumor mechanism of CBD on different types of cancer, there are few clinical trials that have actually applied CBD as a cancer treatment [76]. Kenyon J. et al. [77] undertook a routine analysis of the data collected from 119 cancer patients and reported that about 92% of these patients with solid tumors had good clinical symptoms after treatment with synthetic CBD drugs. Improvements included tumor shrinkage and reduction in the number of circulating cells, and no obvious side effects were identified. Barrie et al. [78] reported a patient with serous ovarian cancer who was treated with a sublingual drop of CBD oil every night for two months, in combination with Laetrile tablets containing mandelic acid. The patient’s follow-up CT results showed a reduction in the bilateral masses and enlarged lymph nodes in the pelvis. Similarly, Sule-Suso et al. [79] reported that the treatment of an elderly patient with lung adenocarcinoma with CBD oil for one month resulted in almost the complete disappearance of the mass in the left lower lobe and a significant decrease in the mediastinal lymph nodes, according to CT scans. These clinical cases of different types of cancer have provided convincing evidence that the use of CBD has significant benefits for tumor patients. The clinical experimental research on CBD still needs further improvement.

## 5. Summary and Prospect

The antitumor effects of CBD in different types of cancer have attracted widespread attention, and the number of relevant research results is steadily increasing. The studies published to date all point to the obvious antitumor effects of CBD and that its mechanisms include the induction of cell cycle arrest and autophagy, the promotion of apoptosis, the modulation of angiogenesis, and the inhibition of tumor cell migration and invasion. In addition, CBD has good synergistic effects with other medicines, and several clinical reports show CBD being used to treat cancer. The results presented in this review indicate that CBD has extremely promising potential for clinical use in the treatment of cancer patients.

CBD is in urgent need of structural optimization due to its structural simplicity, poor water solubility, low bioavailability, and significant inhibition of its antioxidant properties and stability, which limits its clinical applications. It has been confirmed that polymer synthesis, structural modification, and the introduction of hydrophilic groups can enhance the antioxidant properties of CBD and improve its solubility and permeability. Although there are few related studies, this undoubtedly provides a reference for further in vivo experimental studies and clinical applications of CBD, which has a broad application prospect. The antitumor mechanism of CBD appears to be very complex. More detailed and in-depth investigations are needed to provide a stronger theoretical basis for advancing the clinical applications of CBD.
